# Supplementary material for: Influence of Different Stabilization Systems and Multiple Ultraviolet A (UVA) Aging/Recycling Steps on Physicochemical, Mechanical, Colorimetric, and Thermal-Oxidative Properties of ABS
Source: Materials (Basel). 2020 Jan 4;13(1):212. doi: 10.3390/ma13010212 (PMC6981387; doi:10.3390/ma13010212)
Supplement: Supplementary file 1 [file materials-13-00212-s001.pdf]

materials

MDPI

Supplementary Materials

Influence of Different Stabilization Systems and Multiple Ultraviolet A (UVA) Aging/Recycling Steps on Physicochemical, Mechanical, Colorimetric, and Thermal-Oxidative Properties of ABS

Rudinei Fiorio, Sara Villanueva Díez, Alberto Sánchez, Dagmar R. D'hooge and Ludwig Cardon

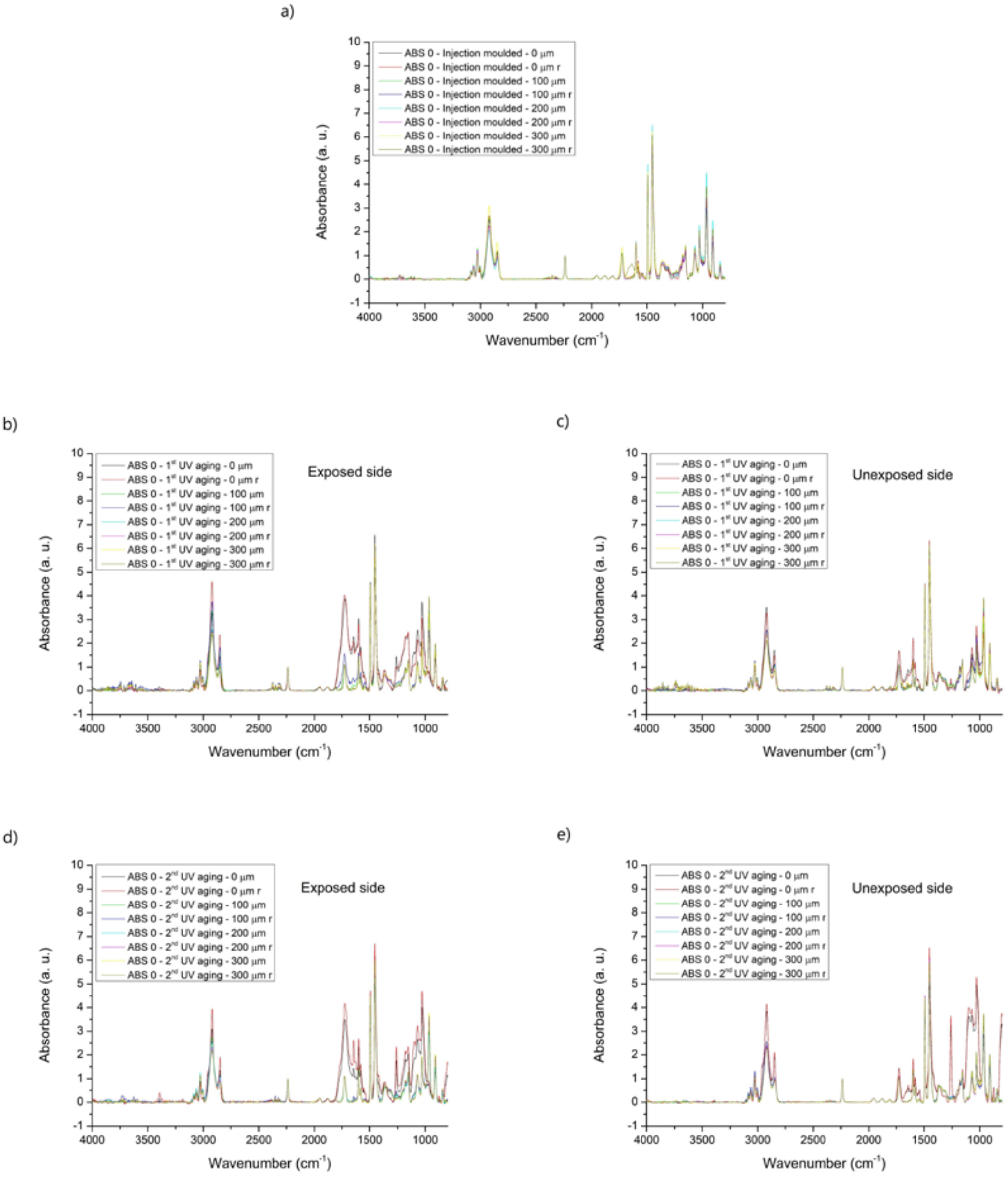

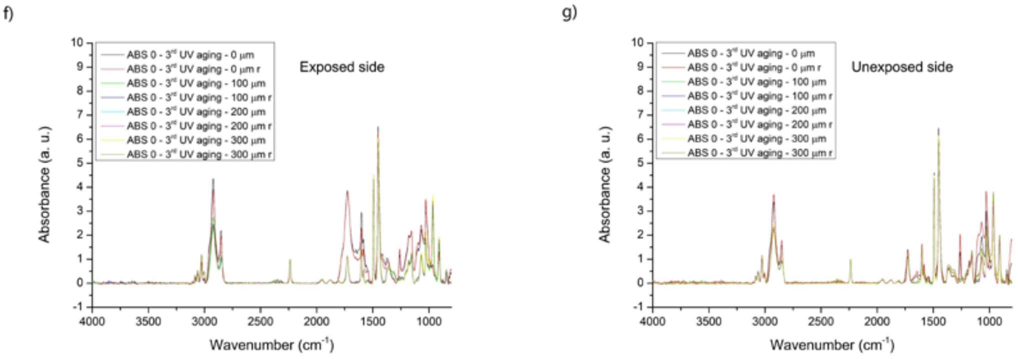

Figure S1. FTIR spectra of the sample type ABS 0. **a**) Injection molded; **b**) and **c**) 1st aging, UV exposed side and unexposed side, respectively; **d**) and **e**) 2nd aging, UV exposed side and unexposed side, respectively; **f**) and **g**) 3rd aging, UV exposed side and unexposed side, respectively. 'r' means repeated analysis.

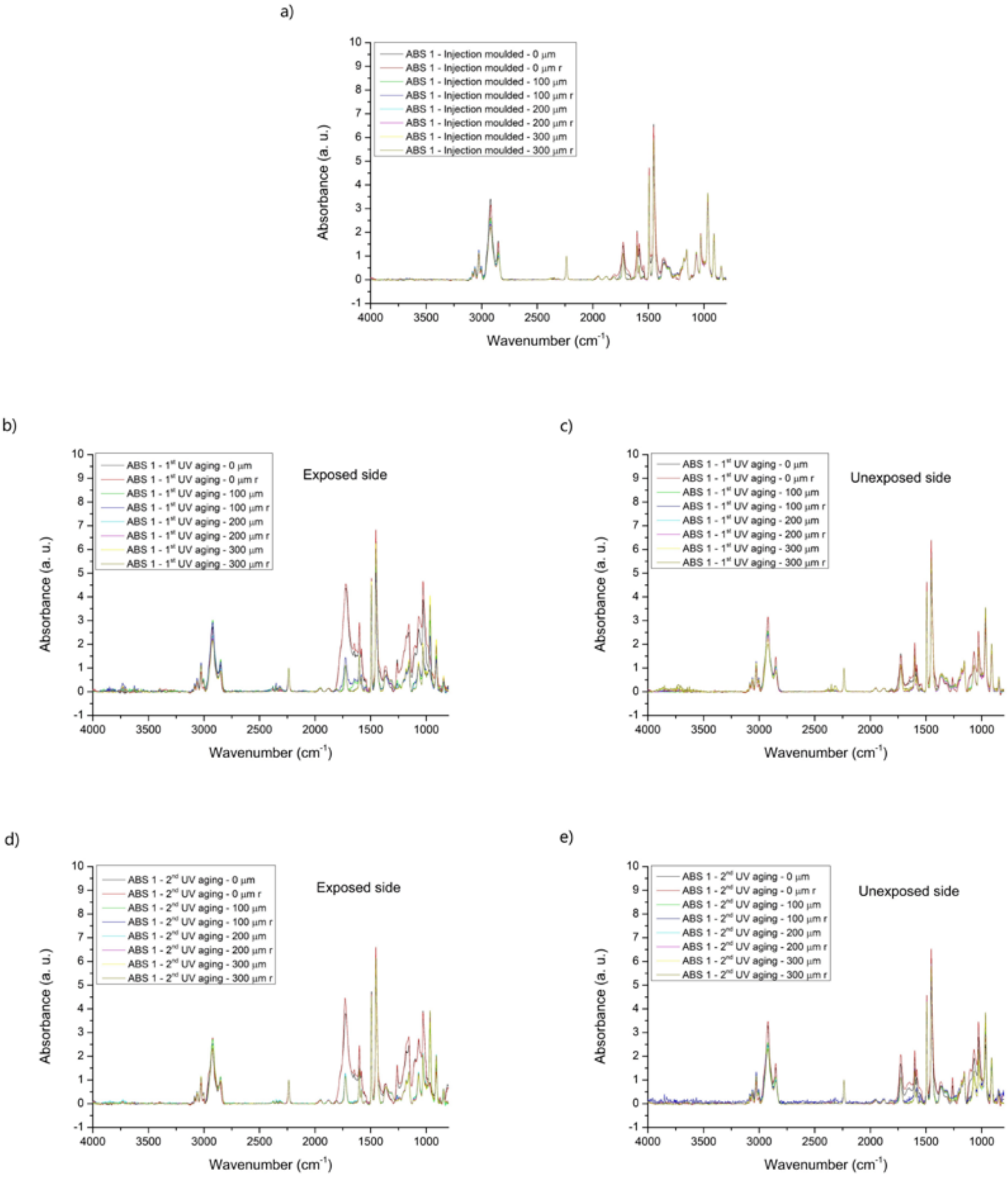

f)

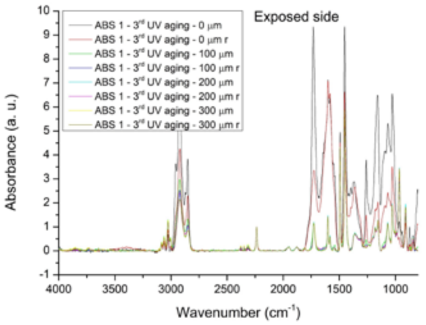

g)

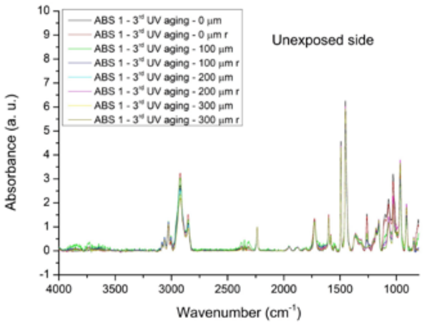

Figure S2. FTIR spectra of the sample type ABS 1. **a**) Injection molded; **b**) and **c**) 1st aging, UV exposed side and unexposed side, respectively; **d**) and **e**) 2nd aging, UV exposed side and unexposed side, respectively; **f**) and **g**) 3rd aging, UV exposed side and unexposed side, respectively. 'r' means repeated analysis.

a)

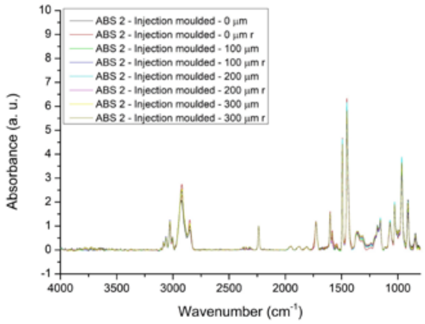

b)

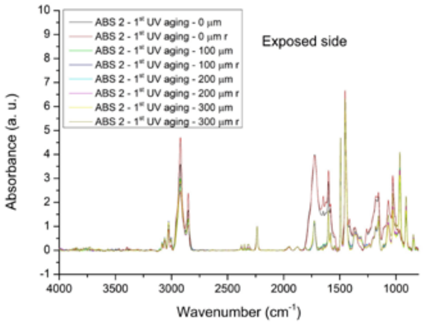

c)

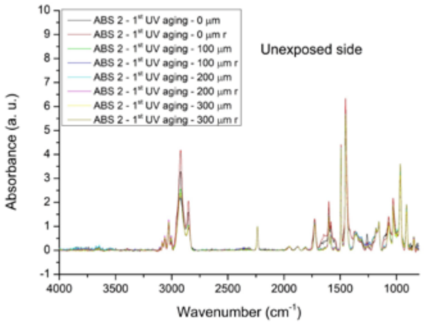

d)

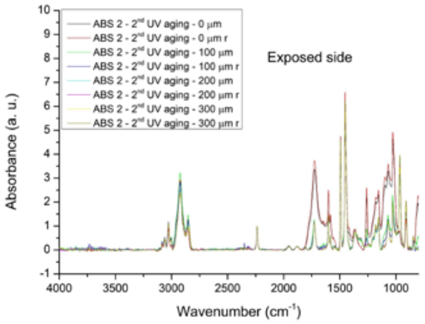

e)

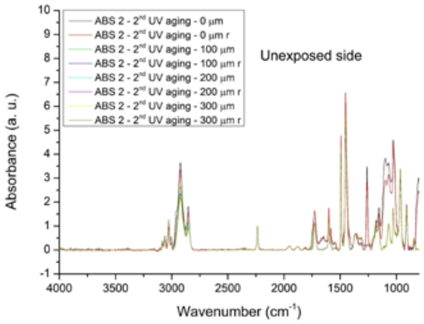

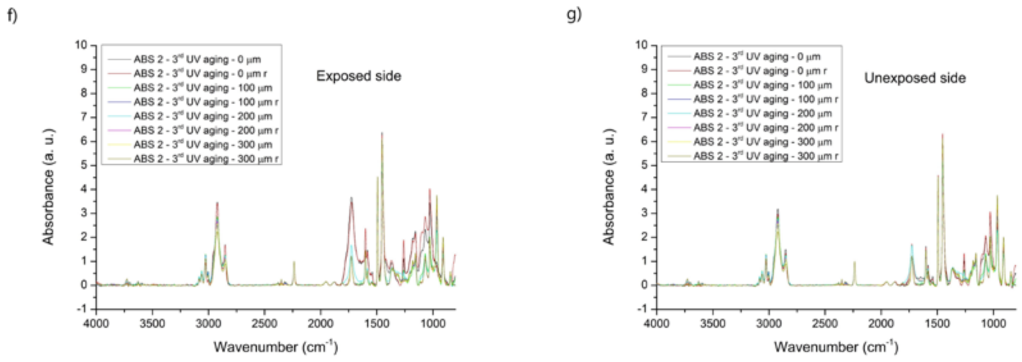

Figure S3. FTIR spectra of the sample type ABS 2. **a**) Injection molded; **b**) and **c**) 1st aging, UV exposed side and unexposed side, respectively; **d**) and **e**) 2nd aging, UV exposed side and unexposed side, respectively; **f**) and **g**) 3rd aging, UV exposed side and unexposed side, respectively. ‘r’ means repeated analysis.

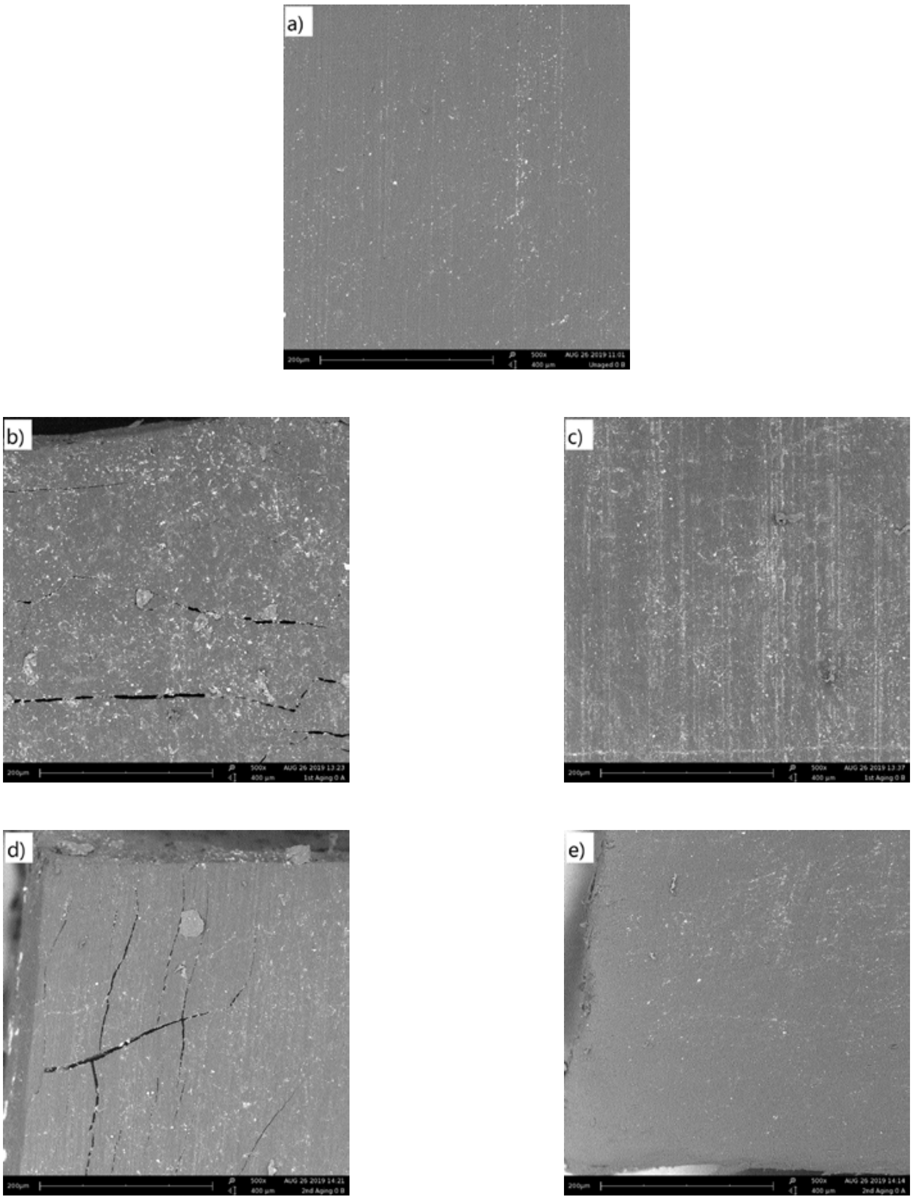

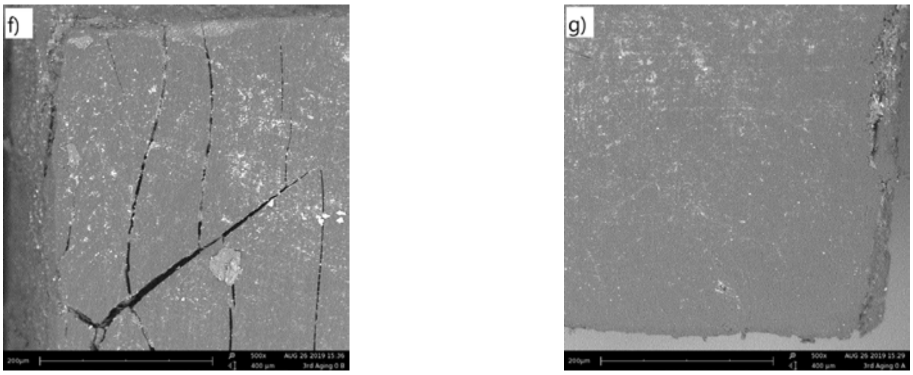

Figure S4. SEM images of the sample type ABS 0. **a**) Injection molded (unaged); **b**) and **c**) 1st aging, UV exposed side and unexposed side, respectively; **d**) and **e**) 2nd aging, UV exposed side and unexposed side, respectively; **f**) and **g**) 3rd aging, UV exposed side and unexposed side, respectively.

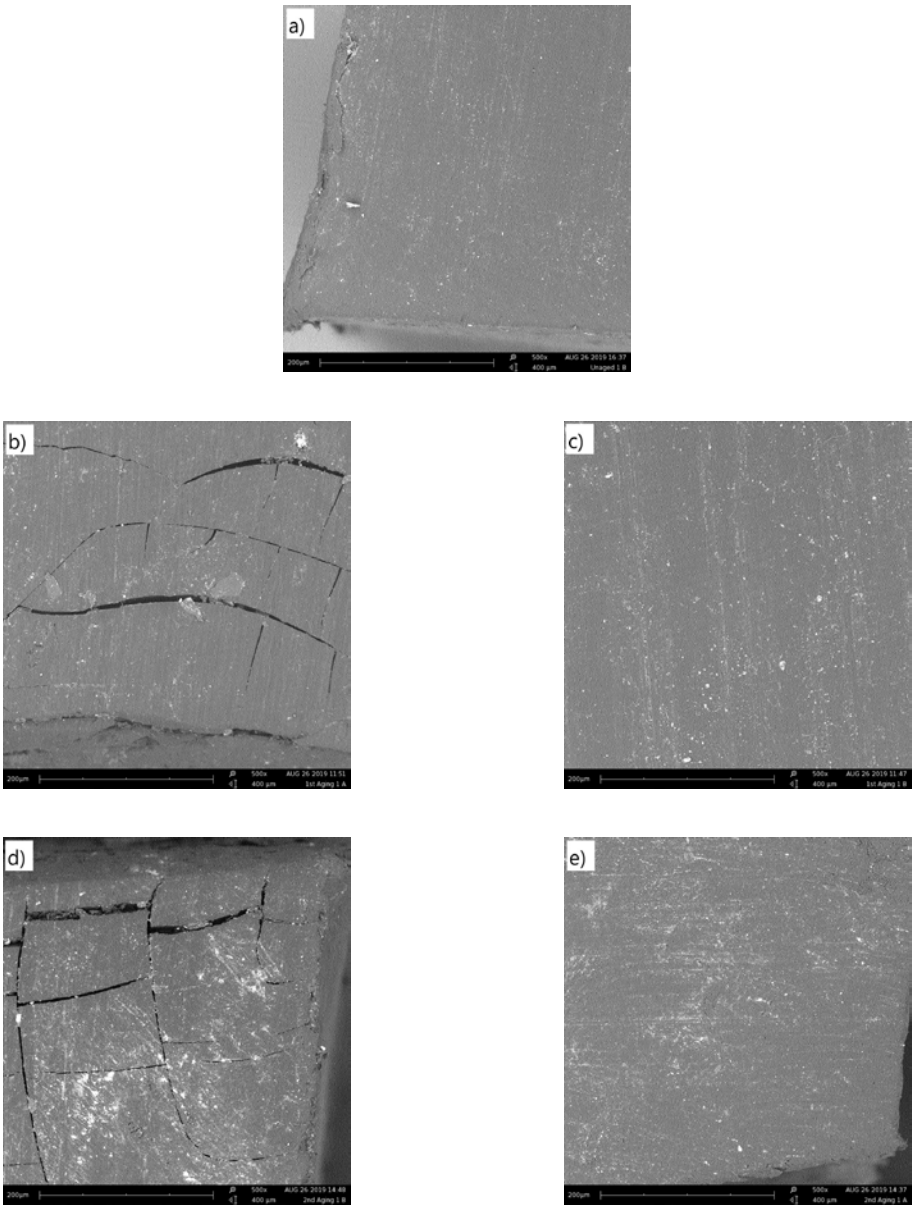

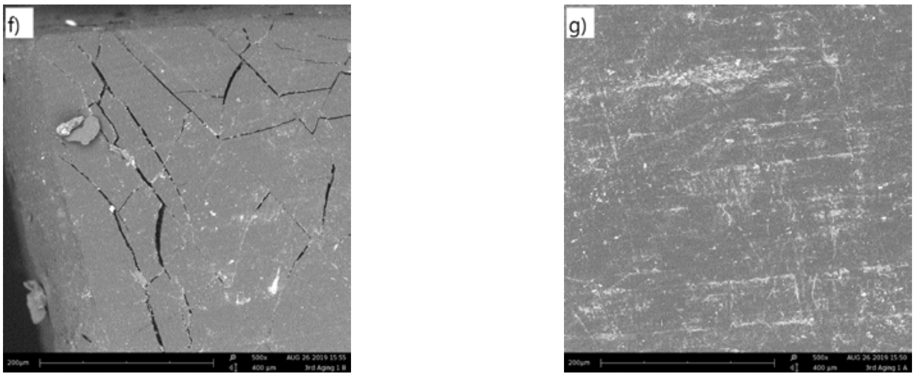

Figure S5. SEM images of the sample type ABS 1. **a**) Injection molded (unaged); **b**) and **c**) 1st aging, UV exposed side and unexposed side, respectively; **d**) and **e**) 2nd aging, UV exposed side and unexposed side, respectively; **f**) and **g**) 3rd aging, UV exposed side and unexposed side, respectively.

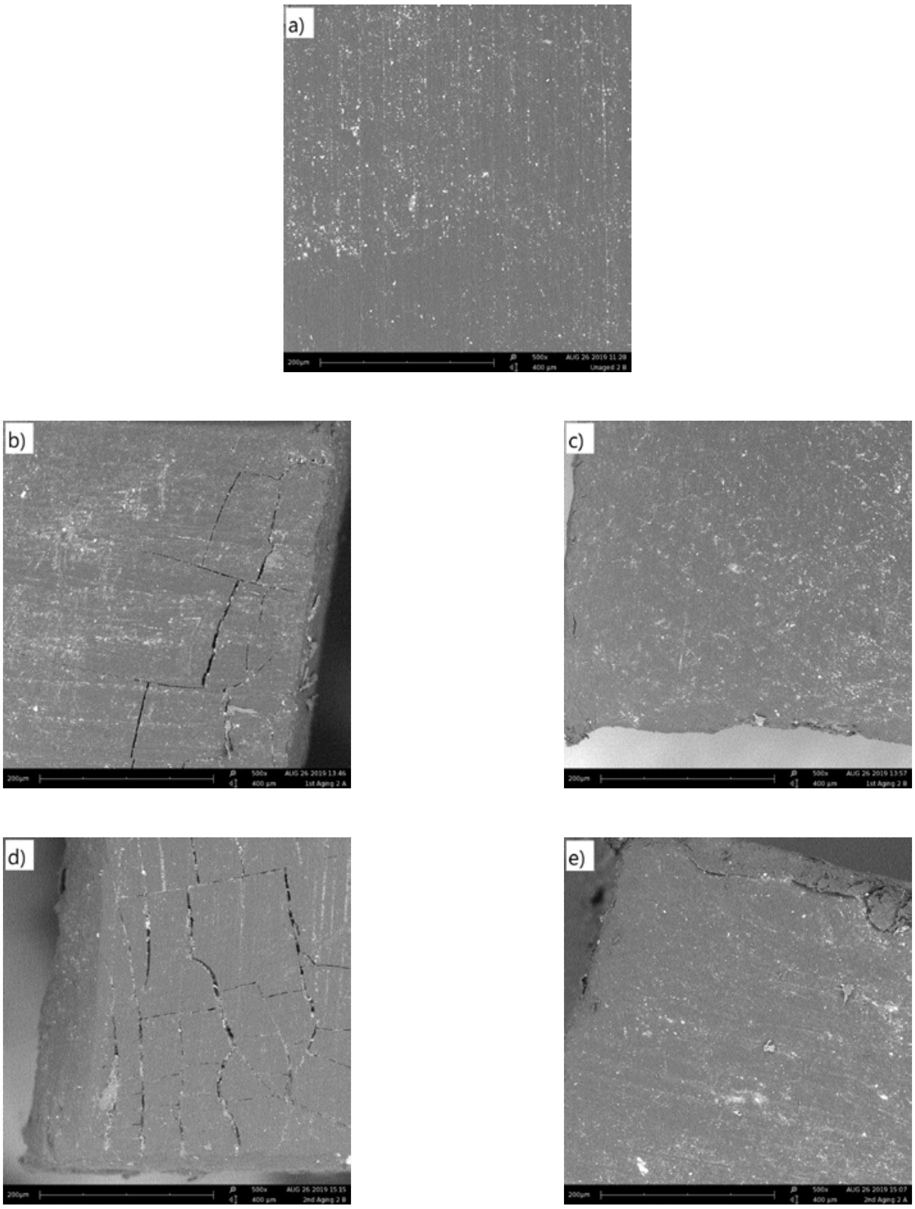

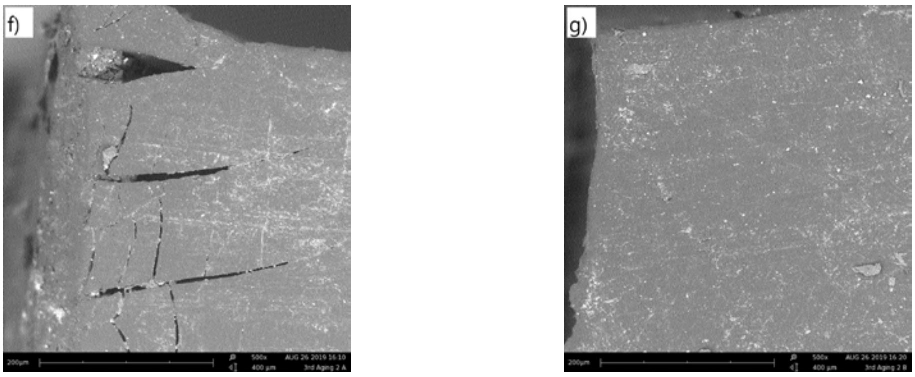

Figure S6. SEM images of the sample type ABS 2. **a**) Injection molded (unaged); **b**) and **c**) 1st aging, UV exposed side and unexposed side, respectively; **d**) and **e**) 2nd aging, UV exposed side and unexposed side, respectively; **f**) and **g**) 3rd aging, UV exposed side and unexposed side, respectively.

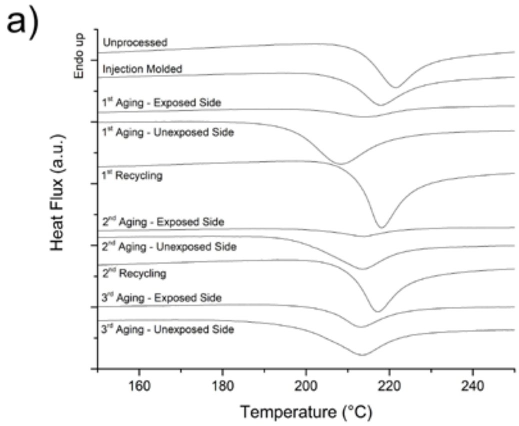

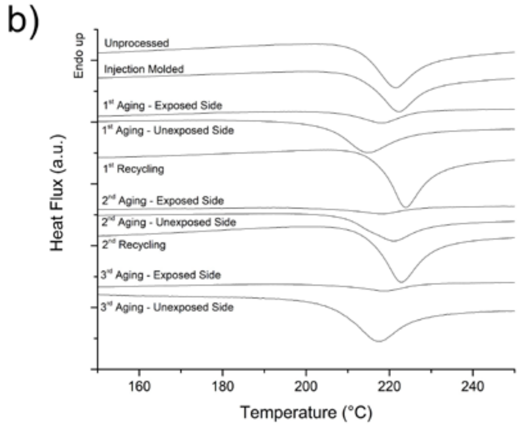

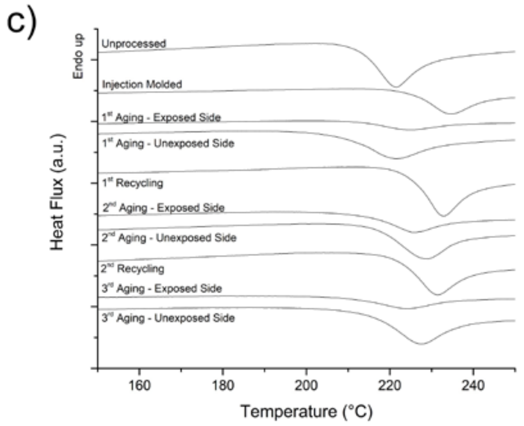

Figure S7. Thermal oxidative resistance of the samples. **a**) Heat flux curves of the sample type ABS 0; **b**) Heat flux curves of the sample type ABS 1; **c**) Heat flux curves of the sample type ABS 2.

© 2020 by the authors. Submitted for possible open access publication under the terms and conditions of the Creative Commons Attribution (CC BY) license (http://creativecommons.org/licenses/by/4.0/).
